# Longitudinal Analysis of Traditional Inflammatory Markers (IL-6, CRP) Juxtaposed With Heparin-Binding Protein (HBP) and Serum Amyloid A Protein Component (SAA) During Acute Infection and Convalescence From COVID-19 Infection in the Context of Initial Viral Load and Markers of Tissue Destruction

**DOI:** 10.1155/jimr/8881752

**Published:** 2025-06-28

**Authors:** Ahmed Sayed Ahmed, Mohamed A. Mahmoud, Hossam Gad, Mohamed Antar, Abdelhamed Elgazar, Vasishta Anil, Daniel A. Diedrich, Krzysztof Laudanski

**Affiliations:** ^1^Pulmonary and Critical Care Division, Mayo Clinic, Rochester, Minnesota, USA; ^2^Department of Anesthesiology and Perioperative Care, Mayo Clinic, Rochester, Minnesota, USA; ^3^Ashyana Consulting, Dundee, Scotland, UK

**Keywords:** acute infection, C-reactive protein, COVID-19, inflammation, post-viral syndrome, SARS-CoV-2

## Abstract

**Purpose:** Characterization of immune system heterogeneity in COVID-19 patients by comparing the inflammatory markers heparin-binding protein (HBP), serum amyloid A protein (SAA), IL-6, and C-reactive protein (CRP) in relation to viral burden, immune response, and tissue damage. Also, determine if prolonged elevations in these markers are associated with long-term symptoms of COVID-19.

**Methods:** This study enrolled 106 hospitalized patients with PCR-confirmed diagnoses of COVID-19. Blood samples were collected within 24 h of admission (*t*_24h_), at 48 h (*t*_48h_), 7 days (*t*_7d_), and long-term, greater than 1 month, post discharge (*t*_LongTerm_). Serum levels of HBP, SAA, IL-6, and CRP were measured using a commercial point-of-care device. Viral burden was assessed via serum viral spike S-protein serum levels and specific immunoglobulins G, M, and D against S&N proteins, and SARS-CoV-2 proteins were quantified. Tissue injury was evaluated by measuring HMGB-1 levels. Clinical data were reviewed retrospectively. Healthy individual samples served as controls.

**Results:** COVID-19 patients exhibited significantly elevated serum HBP and SAA compared to healthy controls. SAA levels normalized over 1 month, whereas HBP, CRP, and IL-6 remained persistently elevated. Patients requiring intensive care unit (ICU) admission, endotracheal intubation, or extracorporeal membrane oxygen (ECMO) demonstrated higher CRP, IL-6, and HBP at initial assessment. However, after 48 h, only IL-6 was elevated in patients who subsequently expired. No significant correlations emerged between serum HBP levels and HMGB-1, viral spike protein levels, or antibodies against SARS-CoV-2 proteins. Pre-existing or acquired comorbidities did not significantly influence HBP or SAA concentrations. An analysis of biomarker profiles identified four distinct patient clusters, each characterized by unique inflammatory patterns that remained stable over time. Specifically, Cluster 1 exhibited low IL-6 with high SAA and CRP. Cluster 2 had low HBP with the highest IL-6. Cluster 3 demonstrated low SAA and CRP levels, and Cluster 4 exhibited robust inflammatory responses across all markers except IL-6.

**Conclusions:** The persistence of inflammatory markers suggests ongoing inflammatory responses post-COVID-19 infection. Significant heterogeneity observed in inflammatory responses upon admission indicates multiple distinct inflammatory phenotypes, which may have implications for clinical outcomes and management strategies.

## 1. Introduction

Immune response heterogeneity significantly influences outcomes in SARS-CoV-2 infections, leading to various inflammatory phenotypes [1, 2]. Reliance on single inflammatory markers can oversimplify disease characterization, underscoring the need to evaluate multiple markers simultaneously [3]. Interleukin-6 (IL-6) and C-reactive protein (CRP) are commonly measured due to their associations with disease severity and prognosis; IL-6 is central to cytokine release syndrome (CRS) and organ failure [4–9], while CRP closely follows disease trajectory and mortality, with up to 86% of COVID-19 patients demonstrating a rapid increase in CRP [7, 10–14]. Individually, these markers have limitations in predicting intensive care unit (ICU) admission or death at 30 days [6, 6, 15–18].

Alternative biomarkers emerged recently, serum amyloid A proteins (SAA), produced by the liver, share acute-phase characteristics with CRP but operates via distinct mechanisms [19, 20]. Elevated SAA levels have been linked with adverse outcomes [3, 21–23]. Heparin-binding protein (HBP), produced by neutrophils is associated with endothelial dysfunction and increased vascular permeability [24–27]. Elevated HBP levels have been documented in severe pneumonia—especially if complicated by sepsis and vasculitis, showing prognostic value in COVID-19 mortality with 79.5% and 84.6% sensitivities at 18 and 35 ng/mL cutoff values [25, 26, 28–30]. Diversification in the origin and function in combinations of these markers may have a greater clinical relevance highlighting the inherent heterogeneity in immune responses [5, 10, 11, 15, 24, 31]. For instance, combining SAA and CRP has proven effective in evaluating disease progression, while pairing SAA with lymphocyte counts provides greater sensitivity in predicting disease severity compared to single-marker evaluations [23]. These examples suggest that analyzing the network of inflammatory cytokines may be more informative for understanding COVID-19 inflammation and infectious processes in general, than focusing on individual molecules alone [32].

Despite its importance, this topic remains understudied. While complex proteomic approaches offer comprehensive insights into the host response to pathogens, they are costly, time-consuming, and require sophisticated analysis. Conversely, selecting a limited number of markers for bedside testing could facilitate rapid diagnosis and inform clinical decision-making. This approach could clarify the timing of resolution during acute inflammatory episodes, which is especially relevant given reports from COVID-19 survivors experiencing cardiovascular and cognitive decline post-infection [6, 33–37]. Although several hypotheses attempt to explain these persistent health issues, unresolved inflammation remains frequently cited as a primary cause [38, 39]. Nonetheless, there is limited longitudinal data verifying persistent elevation of inflammatory markers long after the acute inflammation associated with COVID-19 resolves [6, 38]. This is an important question as several inflammatory markers are implicated in increased risk of chronic illness as they are often symptomatically or etiologically linked to disease onset [31, 39]. For example, SAA deposition is associated with amyloidosis-like diseases, atherosclerosis, and impaired lung and intestinal function [40–42]. CRP is frequently connected to the progression of atherosclerosis, while IL-6 is critical in cachexia and post-ICU metabolic syndromes. Thus, having markers that assess the duration of inflammation following viral infections may help identify patients who are likely to develop long-term COVID-19 symptoms or other long-term complications [41–43].

This study aims to addresses two important gaps in knowledge by comparing novel inflammatory markers (HBP and SAA) against established markers (IL-6 and CRP) to characterize inflammatory phenotypes associated with tissue damage and viral load in COVID-19 [21, 23, 44]. Additionally, the study investigates the timing and dynamics of inflammation resolution, particularly beyond 15 days post-infection [22], with a specific focus on understanding whether HBP demonstrates unique dynamics due to its distinct production, release, and mechanistic pathways [7, 13, 14, 23, 30, 41, 44].

## 2. Methods

### 2.1. Studied Cohort

This single-center study enrolled a convenience sample of patients (*n* = 106) admitted to the hospital with a PCR-positive, acute COVID-19 infection between March and September 2020. None of the enrolled patients had received a COVID-19 vaccine. Demographic and clinical characteristics of the patient cohort are detailed in Table 1. Serum samples obtained from healthy individuals (*n* = 8) served as technical control and reference point. Due to recruitment challenges, the healthy control group was predominantly Caucasian, slightly younger, and mostly male, partially reflecting the demographics of our patient population. However, this group was specifically recruited to ensure technical accuracy and reliability of the results.

### 2.2. Sample Collection

Blood samples were drawn within 24 h of admission (*t*_24h_), then again at 48 h (*t*_48h_), 7 days or at discharge (*t*_7d_), and at long-term – greater than 1-month post-discharge (*t*_>28d_). The distribution of these samples is shown in Figure S1. Blood was collected using Vacutainer tubes (BD, Franklin Lakes, NJ) and immediately put on ice. Plasma was separated by centrifuging the blood at 1000 × g, 10 min at 4°C within 3 h of collection. Plasma aliquots were subsequently stored at −80°C.

### 2.3. Assessment of the Inflammatory Markers, Viral Burden, and Specific Immunological Response

CRP, IL-6, SAA, and HBP were assessed using commercially available platforms according to the manufacturer's guidelines (Joinstar Biomedical Technology Co. Ltd., Hangzhou, People's Republic of China). SARS-CoV-2-specific S-protein was measured using a commercially available kit (RayBiotech, Norcross, GA). Tissue destruction was evaluated by measuring plasma level of HMGB-1 using an ELISA assay (Aviva System Biology, San Diego, CA). Specific immunoglobulin responses against SARS-CoV-2 proteins S&N were analyzed using a commercially available immunoglobulin assay kit (RayBiotech, Norcross, GA).

### 2.4. Collection of Clinical Data

Electronic medical records (EMR) were utilized to collect demographic and clinical information. Acute Physiology and Chronic Health Evaluation II (APACHE II) was calculated at 1 h (APACHE_1 h_) and 24 h following admission (APACHE_24 h_). The Charlson Comorbidity Index (CCI) was used to evaluate the chronic disease burden, while illness severity was assessed using the Sequential Organ Failure Assessment (SOFA). Organ failure was defined using RIFLE criteria or the Glue Grant framework. Diagnoses of deep venous thrombosis (DVT), pulmonary embolism (PE), and stroke were identified from provider notes. Six-month survival was determined post-discharge. Treatments with hydroxychloroquine, remdesivir, convalescent plasma, or steroids were strictly protocolized per hospital policy in line with FDA recommendations during the study period. According to healthcare provider documentation, steroid treatment included any intravenous or oral glucocorticoids administered for COVID-19 pneumonia.

### 2.5. Ethical Approval

This study was conducted in accordance with the guidelines of the Declaration of Helsinki and approved by the Institutional Review Board (IRB) of the University of Pennsylvania (813913). Written informed consent was obtained from all participants before enrollment. Healthy control subjects consented under a separate protocol approved by the IRB of the University of Pennsylvania (815686).

### 2.6. Statistical Analysis

The Shapiro-Wilk W test and distribution plots were used to assess the normality of variable distributions. Parametric variables were expressed as mean ± standard deviation (SD) and compared using *t*-Student (*t*[*n*]). Nonparametric variables were reported as median (Me) with interquartile ranges (IR) and analyzed using the *U*-Mann–Whitney statistic (*U*[dƒ;*n*]). Multiparametric analysis employed ANOVA (ANOVA [dƒ;*n*]) or multiple nonparametric comparisons with Shaffe's post-hoc testing. Longitudinal pairwise comparisons were conducted using the Wilcoxon test (W[*n*]). Correlations were evaluated using Pearson's correlation coefficient (*r*). Clustering analysis was performed using unsupervised *k*-means. A two-sided *p*-value less than 0.05 was considered statistically significant for all tests. Statistical analyses were conducted using Statistica 11.0 (StatSoft Inc., Tulsa, OK) or SPSS (IBM, Armonk, NY). Graphical representations were created using Prisma Graph software (Prisma, Cambridge, MA).

## 3. Results

### 3.1. Characteristics of the Studied Cohort

We enrolled 106 patients whose demographics and clinical characteristics are summarized in Table 1. There was no significant difference in demographic distribution or patient disposition among those providing samples at all four time points (Table 1).

### 3.2. Evolution of Serum Levels of HBP in COVID-19

**Figure 1:**
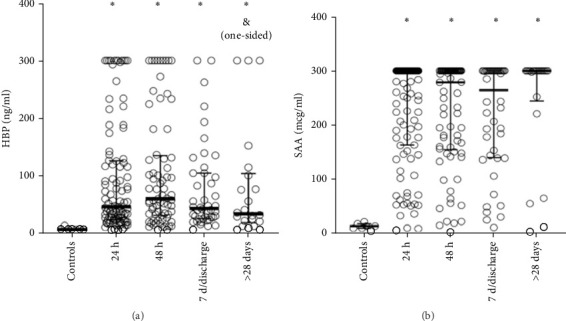
Serum levels of HBP were significantly elevated at all measured time points compared to healthy controls. However, HBP levels measured at 28 days were significantly lower compared to acute admission levels when analyzed using a one-sided hypothesis (A). SAA levels were significantly elevated at 24- and 48-h time points compared to the healthy controls (B). (*⁣*^*∗*^) Indicates a statistical significance when compared to healthy controls (*p* < 0.05). (&) Indicates statistical significance when compared to acute elevation (*p* < 0.05). HBP, heparin-binding protein; SAA, serum amyloid A.

The levels of HBP were significantly elevated at all surveyed time-points compared to the healthy controls. However, following the initial samples at 24 h, subsequent increases were not statistically significant, and serum levels gradually declined, approaching values similar to healthy control values and lower than the initial 24-hour measurement (Figure 1A).

Serum HBP admission levels correlated weakly with length of hospital stay (*r* = 0.24; *p*=0.016) and SOFA scores (*r* = 0.26; *p*=0.008). Patients admitted to the ICU had higher levels of serum HBP during admission compared to non-ICU patients (HBP_ICU_ = 109.9±101.61 vs. HBP_Non-ICU_ = 77.7±89.55, *U* [104] = 2.733; *p*=0.006). Patients requiring mechanical ventilation (MV) had elevated serum levels of HBP at admission (HBP_Vented_ = 131.5±116.82 vs. HBP_Non-Vented_ = 75.1±79.0, *U* [104] = 2.573; *p*=0.01) and at 48 h (HBP_Vented_ = 130.4±109.83 vs. HBP_Non-Vented_ = 84.2±85.51, *U* [72] = 2.21; *p*=0.027). Extracorporeal membrane oxygen (ECMO)-supported patients exhibited elevated HBP levels at admission (HBP_ECMO_ = 163.0±118.45 vs. HBP_Non-ECMO_ = 86.13±91.62, *U* [104] = 2.417; *p*=0.016) and after 7 days (HBP_ECMO_ = 29.00±12.43 vs. HBP_Non-ECMO_ = 88.4±81.77, *U* [44] = −2.270; *p*=0.021). For those patients intubated, these elevated levels persisted at the baseline until the second sampling time. Patients who died within 6 months had significantly elevated HBP levels at admission (Pt _Dead_ = 137.09±112.14 vs. Pt _Alive_ = 78.64±88.13 *U* [94] = −2.819; *p*=0.005), and long-term follow-up more than 28 days (Pt_Dead_ = 105±88.41 vs. Pt_Alive_ = 46.62±79.95 *U* [20] = −2.646; *p*=0.006). No significant differences in admission HBP levels were observed among patients with ESRD, DVT, PE, or AKI. COVID-19-specific treatments did not affect serum HBP levels (data not shown).

There were no meaningful correlations between admission serum HBP levels and levels of HMGB-1, serum S-protein, or the level of immunoglobulins G, M, and D against proteins S&N.

### 3.3. The Level of SAA Protein in the Aftermath of COVID-19

SAA levels were significantly elevated at 24- and 48-h time points compared to control individuals (Figure 1B). However, no significant differences were observed when comparing SAA levels at acute admission to those measured at subsequent paired time points (Figure 1B).

Additionally, serum SAA levels did not differ significantly between deceased versus surviving patients. Patients who required MV or ECMO or between patients admitted to the ICU were compared to those managed on the general medicine floors (Table 2). Finally, there were no significant correlations between SAA levels and HMGB-1, spike protein, or antibodies (IgA, IgM, or IgG) against S&N proteins.

### 3.4. Changes in IL-6 and CRP in Patients With COVID-19

**Figure 2:**
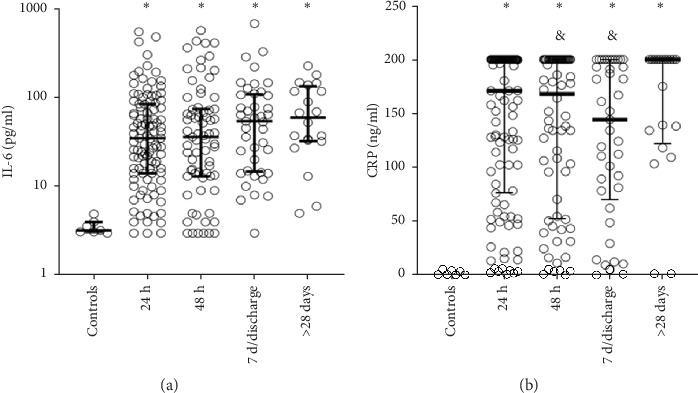
Serum IL-6 levels were significantly elevated in patients diagnosed with COVID-19 compared to healthy controls. However, no significant differences were observed in IL-6 levels across samples drawn at different time points (A). CRP levels were consistently elevated during hospitalization in COVID-19 patients compared to healthy controls. Using the initial admission sample as the reference, CRP levels were significantly lower at 48 h and at 7 days/discharge (&*p* < 0.05) but returned to baseline levels after 28 days (B). (*⁣*^*∗*^) Indicates a statistical significance when compared to healthy controls (*p* < 0.05). (&) Indicates statistical significance when compared to acute elevation (*p* < 0.05). CRP, C-reactive protein; IL-6, interleukin-6.

Serum IL-6 levels were significantly elevated in patients diagnosed with COVID-19 compared to healthy controls. However, no significant changes were observed in IL-6 levels when comparing patient samples collected at two different time points (Figure 2A).

CRP levels were consistently elevated in COVID-19 patients during hospitalization compared to healthy controls. Using the first admission sample as a reference, CRP levels significantly decreased at 24 h and at 7 days post-discharge. However, CRP levels measured after 28 days did not differ significantly from initial admission levels (Figure 2B).

At admission, the SOFA scores significantly correlated with serum IL-6 levels (*r* = 0.43; *p*=0.001) and also showed a significant but weaker correlation with serum CRP levels (*r* = 0.33; *p*=0.001). Patients who died, were admitted to the ICU, required endotracheal intubation, or were placed on ECMO displayed elevated levels of both CRP and IL-6 at admission (Table 2). However, at 48 h following admission, only IL-6 remained significantly elevated among patients who died, were admitted to the ICU, or required endotracheal intubation (Table 2).

Patients with a prior diagnosis of end-stage renal disease (ESRD), DVT, PE, or acute kidney injury (AKI) showed no differences in admission serum CRP or IL-6 levels. Additionally, administration of COVID-19-related treatment did not significantly affect SAA levels.

### 3.5. Complex Analysis of Inflammatory Markers at the Beginning of COVID-19

**Figure 3:**
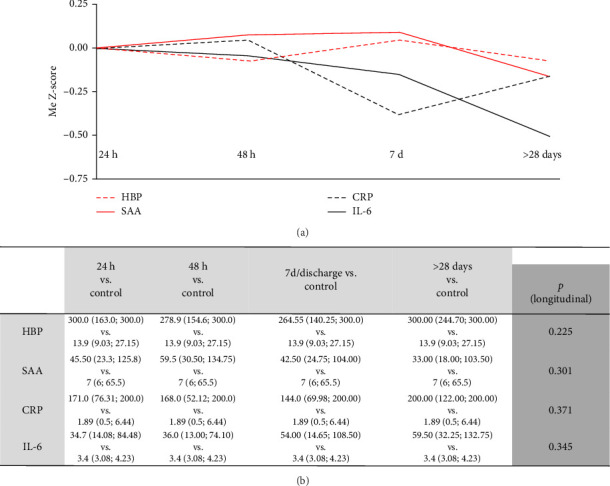
When *Z*-scores of all four markers were plotted together on a common axis, cluster dynamics appeared relatively flat. CRP and IL-6 demonstrated significant declines over time, while HBP and SAA levels remained unchanged (A). Despite these dynamics, all measured markers remained significantly elevated compared to healthy controls (B). CRP, C-reactive protein; HBP, heparin-binding protein; IL-6, interleukin-6; SAA, serum amyloid A.

To compare dynamics of inflammatory markers, serum levels of CRP, IL-6, HBP, and SAA were standardized to Z-scores and plotted on a common axis. The resulting analysis showed relatively flat cluster dynamics, with significant declines observed only in CRP and IL-6, while HBP and SAA remained largely unchanged (Figure 3A,B). At all measured time points, these inflammatory markers remained significantly elevated compared to healthy controls (Figure 3B).

**Figure 4:**
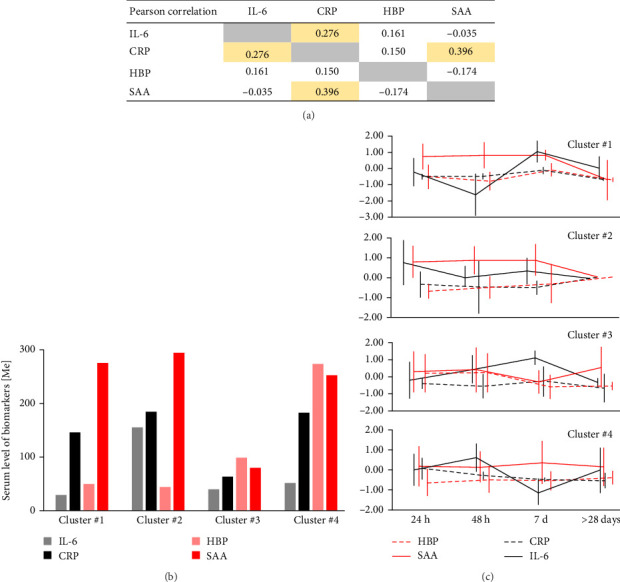
Correlational matrix between studied markers (A). Four clusters were defined using unsupervised *k*-means (B). Clusters' evolution of markers over time (C).

Subsequent cluster analysis was conducted after correlational analysis revealed interdependencies among the studied markers (Figure 4A). Subsequent cluster analysis conducted after correlation analysis revealed interdependencies among the markers studied (Figure 4A). Using unsupervised k-means clustering, four distinct clusters emerged: lowest IL-6 levels with high SAA and CRP levels (cluster #1), lowest HBP levels and highest IL-6 levels (cluster #2), lowest SAA and CRP levels (cluster 33), highest inflammatory response across all markers except IL-6 (cluster #4) (Figure 4B; Figure S1).

Demographic and clinical characteristics of each cluster are detailed in Table 3. Although marker evolution over time differed between clusters, the longitudinal variations did not reach statistical significance (Figure 4C; Table S1). Clinically, clusters differed significantly concerning race, requirement of endotracheal intubation, ECMO, or hospital length of stay (Table 3).

## 4. Discussion

Our study reveals persistent inflammation following COVID-19 with serum trajectories of novel markers (HBP and SAA), demonstrating dynamics similar to traditional markers (CRP and IL-6). Both traditional (IL-6, CRP) and novel (HBP, SAA) markers remained elevated, indicating an ongoing inflammatory response. As demonstrated previously, serum CRP and IL-6 correlated significantly with SOFA scores [7, 11]. Importantly, we observed that serum HBP also correlated with SOFA scores. This finding is consistent with other studies suggesting a relationship between HBP levels and the intensity of viral-host interactions [24–26, 28]. Furthermore, serum HBP was significantly elevated in patients with severe disease, defined as the need for MV or ECMO. It remains unclear if the differences in heparin dosing between ECMO and non-ECMO critically ill patients could influence HBP levels, especially given limited data on the linear relationship between heparin and HBP [45]. Post-COVID-19 serum SAA levels remained persistently elevated and correlated with severity stratification of COVID-19 disease, aligning with prior reports [23]. This prolonged elevation of serum HBP and SAA may contribute to post-COVID-19 complications, including vasculitis and neurological injury as described in other clinical conditions [17, 19, 24, 31, 42].

There was no correlation observed between inflammatory markers and viral load or markers of specific immunity against SARS-CoV-2, suggesting that the studied inflammatory markers reflect a nonspecific inflammatory response rather than specific adaptive immune processes. It is important to note that several patients were admitted relatively late after symptom onset, which could result in immunoglobulin and viral protein levels reaching a plateau or being influenced by ongoing immunological changes.

Our analysis demonstrated significant heterogeneity in immunological responses among COVID-19 patients. Unlike other studies, we employed unsupervised cluster analysis, a data-driven approach that avoids operational bias, to illustrate this heterogeneity [5, 8, 22, 25, 26, 29, 46]. Due to limited sample size, we were unable to examine the evolution of these clusters over time using advanced methods such as the Latent GOLD technique [47]. Nevertheless, we identified distinct patient subgroups with unique marker profiles: lowest IL-6 levels with high SAA and CRP levels (cluster #1), lowest HBP levels and highest IL-6 levels (cluster #2), lowest SAA and CRP levels (cluster #3), highest inflammatory response across all markers except IL-6 (cluster #4). These clusters differed significantly in terms of clinical outcomes, including length of hospital stay, the necessity of ECMO support, and MV, with clusters 2 and 4 showing the most unfavorable clinical outcomes. This finding aligns with prior observations that CRP and HBP are reliable predictors of adverse outcomes [7, 26, 28, 29]. A clear correlation between IL-6 and HBP was evident only in Cluster 2, suggesting this subgroup represents a distinct population characterized by classical inflammatory markers. Additionally, race emerged as a differentiating factor among clusters, potentially reflecting underlying socioeconomic determinants of health influencing cluster assignment [11, 18, 36]. Specific changes in markers triggered by COVID-19 infection could not be definitively confirmed due to the absence of a control population in our analysis, but our identified clusters were based on a relatively small sample size [6, 32].

Our study also explored the use of a point-of-care device as bedside tools to support patient management. In our experience, the device's dynamic range significantly impacted its performance. Specifically, serum SAA frequently exceeded the measurable range of the device due to high baseline levels further elevated during acute illness [19, 23]. Although sample dilution could theoretically resolve this issue, this step adds an unrealistic burden in clinical practice. Aside from this limitation, the device was previously validated and demonstrated reliable performance [48]. Additionally, the interaction between HBP and heparin should be carefully considered during sample collection to ensure accurate measurements [45, 48, 49].

The methodological limitations of the study included selection bias, as the less severely ill patients were more likely to drop out during follow-up. Although our cohort size was modest, it was comparable to other early COVID-19 studies. However, limited sample size constrained the robustness of the *k*-clustering analysis, and these findings should be considered preliminary. Future studies will include larger cohorts to improve the statistical power and validity of the clustering results. The absence of prior reference data for SAA and HBP complicated power calculations making our cohort more akin to a convinience sample. The control group was also small; but its primary purpose was to provide baseline measurements from healthy individuals negative for the markers under investigation. Longitudinal analysis helped mitigate patient heterogeneity, but socioeconomic determinants, particularly the predominance of African-American patients with higher mortality rates, introduced further bias. Our healthy control group was selected to show technical accuracy of the assessments, not being a straighforward comparison to studied individuals. In the latter case we would have to follow healthy individuals longitudinally as seem in the control group. Retention of sicker patients with prolonged inflammatory responses may explain the persistent elevation of inflammatory markers, even after acute inflammation subsided. Our study did not differentiate between various SAA isoforms, which some research suggests could vary in predominance depending on pathogen type and influence inflammatory responses differently [17, 50, 51]. Current technology to distinguish these isoforms remains labor-intensive and impractical for clinical settings [50]. Additionally, we did not collect data on illicit drug use, which may influence inflammatory markers. Lastly, all patients received varying doses of heparin, potentially affecting HBP levels unpredictably, thus complicating interpretation of these results [45, 48, 49].

Our study presents several unique advantages. First, we enrolled a predominantly African-American cohort with a high burden of pre-existing illnesses, accurately representing a particularly vulnerable population. Additionally, since our sample was drawn from patients affected during the initial COVID-19 wave, the findings are unaffected by pre-existing immunological memory from prior SARS-CoV-2 infections. Lastly, the longitudinal design of our study provides distinct insights into the dynamics of inflammatory responses over time, which remains relatively uncommon in COVID-19 research [38].

This study demonstrated that serum levels of HBP and SAA were significantly elevated in patients diagnosed with COVID-19. While serum SAA levels normalized over time, HBP, CRP, and IL-6 remained persistently elevated even after 1 month. Elevated levels of HBP, CRP, and IL-6 correlated strongly with adverse clinical outcomes, including ICU admission, endotracheal intubation, ECMO support, and increased mortality. Conversely, SAA did not show a significant correlation with clinical outcomes. We identified four distinct patient clusters. These clusters differed significantly regarding race, severity of illness, and length of hospital stay: Cluster 2 and 4 were characterized by elevated IL-6 and HBP levels, respectively, and were associated with the most unfavorable outcomes. Cluster 1 and 3 had lower IL-6 and HBP levels, respectively, and demonstrated better outcomes. Our findings indicate that HBP and SAA could serve as valuable markers to identify specific inflammatory phenotypes and potentially predict long-term complications associated with COVID-19. Furthermore, the study confirmed the feasibility of measuring these markers using point-of-care devices, although challenges such as limited dynamic range were noted. The study's conclusions are limited by modest sample size, selection bias, lack of controls for heparin exposure, and absence of differentiation among SAA isoforms. Future studies with larger cohorts and controlled methodologies are needed to validate and expand upon these preliminary results.

## Figures and Tables

**Table 1:** Demographic and clinical characteristics of the enrolled cohort with division for subsequent sampling times.

Characteristics	*t*_+24 h_ (*n* = 106)	*t*_+48 h_ (*n* = 75)	*t*_7d_ (*n* = 45)	*t*_>28d_ (*n* = 23)
Age (X ± SD)	58.9 ± 18.23	59.2 ± 18.07	61.2 ± 16.4	54.8 ± 17.07
Over 60 (%)	56.6%	60.%	62.2%	34.8%
BMI (X ± SD)	31.7 ± 8.91	31.3 ± 7.6	30.2 ± 6.8	29.9 ± 7
Male (%)	60.4%	61.33	44.44	73.91
Race				
Caucasian/Hispanic Latino (%)	26.73%	25.33	31.11	34.78
Black (%)	63.37%	62.67	57.78	43.47
Other/Asian/unknown (%)	9.9%	12	11.11	21.73
ICU (%)	50%	62.67	75.55	65.22
Intubated (%)	33%	40	57.78	60.87
ECMO (%)	9.4%	12	20	39.13
Length of stay (X ± SD)	17.51 ± 26.122	23.15 ± 29.19	33.16 ± 34.01	44.83 ± 42.9
Length of stay in the ICU (X ± SD)	10.92 ± 24.533	14.86 ± 27.89	23.27 ± 33.34	33.05 ± 43.47
APACHE SCORE 1 HR (X ± SD)	11.13 ± 7.809	11.57 ± 7.7	13.6 ± 8.09	12.91 ± 8.08
APAHE SCORE 24 HR (X ± SD)	11.04 ± 7.313	11.73 ± 7.18	14.11 ± 7.13	13.26 ± 7.78
Pre-existing conditions				
Myocardial infarction (%)	5.7%	4	2.22	4.35
Congestive heart failure (%)	15.1%	13.33	15.56	21.74
Peripheral vascular disease (%)	7.5%	9.33	11.11	8.70
Cerebrovascular stroke/Transient ischemic Attacks (%)	11.3%	13.33	17.78	13.04
Dementia (%)	3.8%	4	6.66	0.00
Chronic obstructive pulmonary disease (%)	14.2%	16	20	13.04
Connective tissue disease (%)	2.8%	4	6.66	0.00
Peptic ulcer disease (%)	2.8%	2.67	4.44	4.35
Liver disease (%)	1.9%	1.33	2.22	0.00
Diabetes mellitus (%)	34.9%	40	40	43.48
Hemiplegia (%)	3.8%	5.33	8.89	4.35
Chronic kidney disease (%)	24.5%	25.33	31.11	17.39
End stage renal disease (%)	1.9%	2.67	4.44	0.00
Solid tumor (%)	10.4%	8	8.89	13.04
Leukemia %)	0.9%	1.33	2.22	4.35
Lymphoma (%)	0%	0	0	0.00
Acquired immunodeficiency syndrome (%)	0.9%	1.33	2.22	0.00
Smoking status				
Smoker (%)	9.43%	8	6.66	8.70
Former smoker (%)	32.08%	37.33	33.33	21.74
Nonsmoker (%)	58.49%	54.67	60	69.57

**Table 2:** Relationship between clinical trajectory and measured markers over time.

Characteristics		<24 h	48 h	7 d/Discharge	>28d
CRP	Dead vs alive	**200.0 [133.0; 200.0] vs 152.05 [55.03; 200.0]; *p*=0.043**	200.0 [143.0; 200.0] vs 134.0 [43.5; 200.0]; *p*=0.073	144.00 [62.0; 200.0] vs 156.5 [87.00; 194.75]; *p*=0.864	200.0 [115.25; 200.0] vs 187.5 [123.0; 200.0]; *p*=0.735
	ICU vs non-ICU	**200.0 [147.0; 200.0] vs 126.0 [49.0; 200.0]; *p*=0.004**	176.15 [95.78; 200.0] vs 143.0 [31.0; 200.0]; *p*=0.320	156.5 [80.24; 200.0] vs 101.0 [48.52; 193.0]; *p*=0.6	200.0 [134.0; 200.0] vs 139.0 [128.0; 200.0]; *p*=0.696
	Vented vs non vented	**129.2 [55.04; 200.0] vs 200.0 [194.25; 200.0]; *p* < 0.001**	200.0 [194.25; 200.0] vs 140.5 [42.75; 200.0]; *p*=0.129	167.0 [101.0; 200.0] vs 99.5 [11.5; 194.75]; *p*=0.103	200.0 [127.75; 200.0] vs 169.5 [133.0; 200.0]; *p*=0.833
	ECMO vs no ECMO	**200.0 [199.83; 200.0] vs 160.0 [69.19; 200.0]; *p*=0.041**	185.66 [164.0; 200.0] vs 147.31 [50.71; 200.0]; *p*=0.288	193.0 [112.0; 200.0] vs 129.0 [70.6; 191.5]; *p*=0.167	200.0 [175.0; 200.0] vs 139.0 [113.5; 200.0]; *p*=0.261

IL-6					
	Dead vs alive	**88.5 [38.07; 151.0] vs 23.1 [10.43; 55.25]; *p* < 0.001**	**74.1 [28.00; 262.00] vs 24.1 [5.00; 60.00]; *p*=0.002**	59.10 [27.30; 115.00] vs 40.00 [14.75; 84.75]; *p*=0.246	68.00 [40.75; 137.25] vs 35.50 [28.25; 132.75]; *p*=0.354
	ICU vs non-ICU	**48.0 [18.5; 100.65] vs 24.0 [12.0; 57.4]; *p*=0.023**	**56.0 [14.0; 139.1] vs 24.5 [10.75; 44.3];** *p*=0.021	60.05 [18.75; 117.75] vs 31.0 [14.0; 64.0]; *p*=0.270	89.0 [37.0; 138.0] vs 34.0 [32.5; 53.0]; *p*=0.303
	Vented vs non vented	**53.85 [30.73; 121.8] vs 24.25 [12.5; 65.25]; *p*=0.002**	**67.05 [33.25; 207.0] vs 15.5 [5.0; 51.5]; *p* < 0.001**	63.5 [27.3; 115.0] vs 28.0 [11.5; 67.0]; *p*=0.054	**79.0 [36.0; 139.5] vs 35.5 [30.75; 79.5]; *p*=0.028**
	ECMO vs no ECMO	**109.0 [52.725; 153.0] vs 28.65 [14.0; 71.5]; *p*=0.008**	58.0 [39.0; 74.1] vs 29.5 [12.625; 72.75]; *p*=0.169	59.1 [45.0; 75.0] vs 44.5 [14.0; 111.75]; *p*=0.529	89.0 [52.0; 144.0] vs 34.0 [29.5; 93.0]; *p*=0.080

					
HBP	Dead vs alive	**92 [39.75; 300] vs 37 [19.5; 110.5]; *p*=0.005**	66 [45.0; 181.0] vs 55.5 [19.5; 130.0]; *p*=0.319	41.5 [25.25; 127.25] vs 46 [24.75; 102.25]; *p*=0.814	**75.5 [43.75; 142.5] vs 21 [10.5; 33.0]; *p*=0.008**
	ICU vs non-ICU	**72.0 [35.0; 135.5] vs 32.0 [18.0; 115.0] *p*=0.006**	66.0 [39.0; 181.0] vs 47.0 [17.0; 127.0]; *p*=0.104	39.5 [24.75; 109.75] vs 54.5 [31.25; 99.5]; *p*=0.889	47.5 [21.75; 142.5] vs 30.5 [18.0; 45.0]; *p*=0.356
	Vented vs non vented	**65.5 [33.0; 285.75] vs 37.0 [21.25; 110.5]; *p*=0.01**	**81.5 [47.5; 245.75] vs 51.5 [19.0; 123.5]; *p*=0.027**	38.0 [23.25; 78.75] vs 54.5 [33.25; 111.5]; *p*=0.219	55.0 [24.0; 114.0] vs 31.0 [12.0; 40.0]; *p*=0.332
	ECMO vs non ECMO	**132.5 [59.25; 291.0] vs 42.5 [22.0; 118.25]; *p*=0.016**	66.0 [57.0; 181.0] vs 56.0 [ 24.5; 130.0]; *p*=0.287	**29.0 [22.0; 37.0] vs 55.0 [27.5; 122.5]; *p*=0.023**	40.0 [24.0; 76.0] vs 31.0 [12.0; 100.0]; *p*=0.894

SAA	Dead vs alive	281.9 [208.5; 300.0] vs 300.0 [145.0; 300.0]; *p*=0.826	278.8 [167.0; 300.0] vs 260.50 [145.8; 300.0]; *p*=0.983	264.05 [146.25; 300] vs 299.50 [148.75; 300.0]; *p*=0.739	300.0 [236.8; 300.0] vs 300.0 [123.75; 300.0]; *p*=0.929
	ICU vs non-ICU	283.8 [198.5; 300.0] vs 300.0 [145.75; 300.0]; *p*=0.804	262.0 [155.25; 300.0] vs 300.0 [157.5; 300.0]; *p*=0.541	264.55 [146.25; 300.0] vs 265.5 [88.45; 300.0]; *p*=0.748	300.0 [236.8; 300.0] vs 300.0 [300.0; 300.0]; *p*=0.25
	Vented vs non vented	281.9 [212.75; 300.0] vs 300.0 [1 145.0; 300.0]; *p*=0.686	293.0 [195.525; 300.0] vs 246.15 [124.75; 300.0]; *p*=0.103	264.55 [186.25; 300.0] vs 252.9 [64.75; 300.0]; *p*=0.364	300.0 [298.275; 300.0] vs 300.0 [182.0; 300.0]; *p*=0.563
	ECMO vs no ECMO	291.9 [251.275; 300.0] vs 300.0 [162.5; 300.0]; *p*=0.594	286.0 [197.1; 300.0] vs 278.8 [156.0; 300.0]; *p*=0.829	184.0 [153.0; 300.0] vs 295.3 [137.5; 300.0]; *p*=0.716	300.0 [252.6; 300.0] vs 300.0 [297.7; 300.0]; *p*=0.968

*Note:* Bold values indicate statistical significance.

**Table 3:** Changes in clinical characteristics across biomarkers clusters.

Characteristics	Cluster 1 (*N* = 49)	Cluster 2 (*N* = 15)	Cluster 3 (*N* = 22)	Cluster 4 (*N* = 15)	Significance
Age (X±SD)	62.1 ± 18.06	59.8 ± 17.25	55.4 ± 18.87	53.5 ± 17.16	ns
Over 60 (%)	67.3	53.3	50.0	40.0	
BMI (X±SD)	29.9 ± 17.11	31.9 ± 7.28	35.6 = 9.87	34.3 ± 12.21	
Male (%)	57.1	66.7	59.1	60.0	
Race					
Caucasian/Hispanic Latino (%)	20.4%	40.0%	31.8%	26.6%	**0.007**
Black (%)	73.5%	33.3%	68.2%	53.3%	
Other/Asian/unknown (%)	6.1%	26.7%	0.02%	20.1%	
ICU (%)	46.9%	53.3%	53.3%	60.0%	ns
Intubated (%)	26.5%	40.0%	13.6%	60.0%	**0.018**
ECMO (%)	4.1%	20.0%	0.0%	26.7%	**0.009**
Length of stay (X±SD)	11.7 ± 12.96	23.1 ± 26.69	6.1 ± 7.12	26.5 ± 42.15	**0.005**
Length of stay in the ICU (X±SD)	6.07 ± 12.61	23.4 ± 36.49	1.9 ± 3.88	22.2 ± 42.83	ns
APACHE SCORE 1 HR (X±SD)	9.9 ± 7.61	12.0 ± 7.15	7.6 ± 4.58	10.5 ± 7.00	
APAHE SCORE 24 HR (X±SD)	10.4 ± 6.44	11.7 ± 9.11	7.3 ± 5.28	10.7 ± 6.50	
Pre-existing conditions					
Myocardial infarction (%)	4.1%	6.7%	9.1%	0.0%	ns
Congestive heart failure (%)	18.4%	13.3%	4.5%	6.7%	
Peripheral vascular disease (%)	8.2%	0.0%	4.5%	0.0%	
Cerebrovascular stroke/Transient ischemic attacks (%)	16.3%	6.7%	4.5%	0.0%	
Dementia (%)	8.2%	0.0%	0.0%	0.0%	
Chronic obstructive pulmonary disease (%)	18.4%	13.3%	9.1%	0.0%	
Connective tissue disease (%)	6.1%	0.0%	0.0%	0.0%	
Peptic ulcer disease (%)	4.1%	0.0%	4.5%	0.0%	
Liver disease (%)	0.0%	0.0%	9.1%	0.0%	
Diabetes mellitus (%)	30.6%	26.7%	31.8%	53.3%	
Hemiplegia (%)	4.1%	13.3%	0.0%	0.0%	
Chronic kidney disease (%)	22.4%	26.7%	18.2%	26.7%	
End stage renal disease (%)	2.0%	0.0%	0.0%	0.0%	
Solid tumor (%)	14.3%	0.0%	18.2%	0.0%	
Leukemia (%)	0.0%	0.0%	0.0%	0.0%	
Lymphoma (%)	0.0%	0.0%	0.0%	0.0%	
Acquired immunodeficiency syndrome (%)	2.0%	0.0%	0.0%	0.0%	
Smoking status					
Smoker (%)	34.69%	40.0%	36.4%	13.3%	ns
Former smoker (%)	10.2%	6.7%	13.6%	6.7%	
Nonsmoker (%)	55.1%	53.3%	50.0%	80.0%	

*Note:* Bold values indicate statistical significance.

## Data Availability

The datasets used and/or analyzed during the current study are available from the corresponding authors upon reasonable request and after approval by the IRB.

## Supporting Information

Supporting Information After performing cluster analysis on the correlational matrix, four clusters were identified using unsupervised k-means clustering, resulting in a total of four distinct clusters. Cluster #1 exhibited the lowest levels of IL-6 and high levels of SAA and CRP. Cluster #2 demonstrated the lowest level of HBP and the highest level of IL6. Cluster #3 had the lowest levels of SAA and CRP. Cluster 4 exhibited the most robust inflammatory response across all markers except for IL-6. The longitudinal analysis of serum markers at each cluster time point revealed no statistically significant differences. In some statistical contrast, few cases precluded meaningful statistical analysis.
