# Supplementary material for: Longitudinal Analysis of Traditional Inflammatory Markers (IL-6, CRP) Juxtaposed With Heparin-Binding Protein (HBP) and Serum Amyloid A Protein Component (SAA) During Acute Infection and Convalescence From COVID-19 Infection in the Context of Initial Viral Load and Markers of Tissue Destruction
Source: J Immunol Res. 2025 Jun 28;2025:8881752. doi: 10.1155/jimr/8881752 (PMC12228567; doi:10.1155/jimr/8881752)
Supplement: Supporting Information — After performing cluster analysis on the correlational matrix, four clusters were identified using unsupervised k-means clustering, resulting in a total of four distinct clusters. Cluster #1 exhibited the lowest levels of IL-6 and high levels of SAA and CRP. Cluster #2 demonstrated the lowest level of HBP and the highest level of IL6. Cluster #3 had the lowest levels of SAA and CRP. Cluster 4 exhibited the most robust inflammatory response across all markers except for IL-6. The longitudinal analysis of serum markers at each cluster time point revealed no statistically significant differences. In some statistical contrast, few cases precluded meaningful statistical analysis. [file 8881752.f1.pdf]

Supplemental Material #1

After performing cluster analysis on the correlational matrix, four clusters were identified using unsupervised k-means clustering, resulting in a total of four distinct clusters. Cluster #1 exhibited the lowest levels of IL-6 and high levels of SAA and CRP. Cluster #2 demonstrated the lowest level of HBP and the highest level of IL6. Cluster #3 had the lowest levels of SAA and CRP. Cluster 4 exhibited the most robust inflammatory response across all markers except for IL-6.

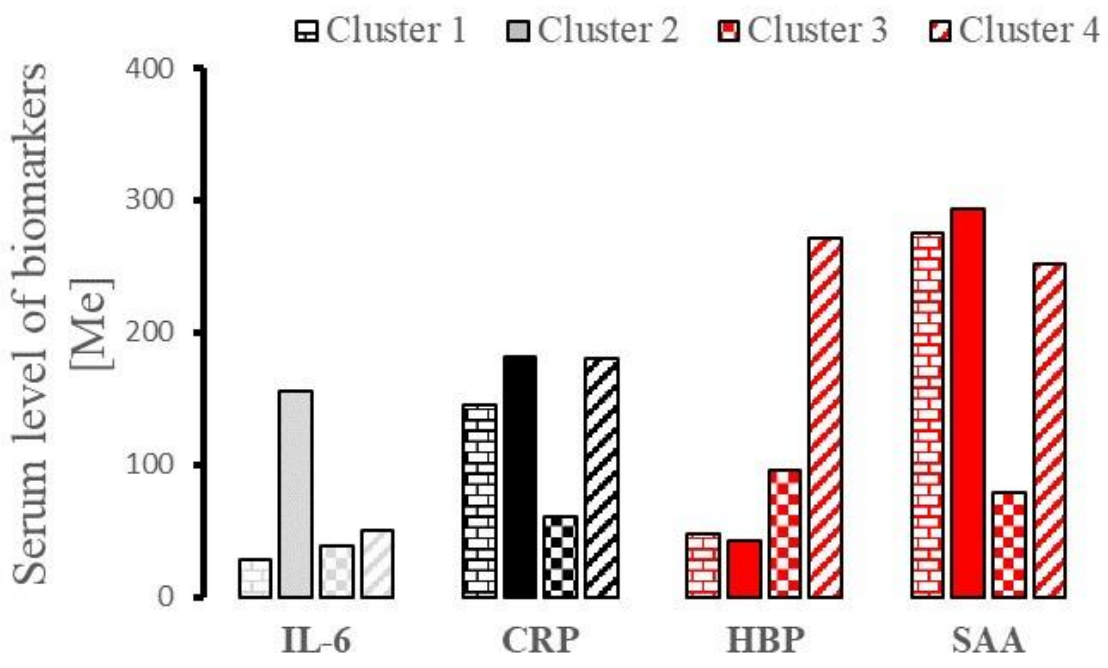

Supplemental Material #2

The longitudinal analysis of serum markers at each cluster time point revealed no statistically significant differences. In some statistical contrast, few cases precluded meaningful statistical analysis.

		**48hrs vs 24hrs**	**7d/discharge vs 24hrs**	**<28 days vs 24hrs**	**p longitudinal**
**Cluster #1**	**HBP**	62.50 [34.75;181.00] *vs.* 54.50 [31.25;186.75]	43.00 [23.50;133.00] *vs.* 54.50 [31.25;186.75]	29.00 [14.25;263.00] *vs.* 54.50 [31.25;186.75]	.488
	**SAA**	286.5 [159.00;300.00] *vs.* 300.0 [197.88;300.00]	243.5[138.25;300.00] *vs.* 300.0 [197.88;300.00]	300.00[298.85;300.00] *vs.* 300.0 [197.88;300.00]	.0.92
	**CRP**	179.19 [40.50;200.00] vs 175.74 [102.10;200.0]	157.90 [83.00;200.00] vs 175.74 [102.10;200.0]	200.0 [175.00;200.00] vs 175.74[102.10;200.00]	.109
	**IL-6**	36.00 [8.50;105.50] vs 30.65 [14.00;91.13]	64.00 [14.15;128.50] vs 30.65 [14.00;91.13]	89.00 [33.00;138.00] vs 30.65 [14.00;91.13]	.207
**Cluster #2**	**HBP**	53.00 [19.50;100.00] vs 26.00 [21.00;67.00]	50.50 [26.00;136.50] vs 26.00 [21.00;67.00]	No Data	.275
	**SAA**	300.0[221.10;300.00] vs 300.0 [246.00;300.00]	300.0[179.35;300.00] vs 300.0 [246.00;300.00]	No Data	.710
	**CRP**	134.0[102.00;200.00] vs 194.0[22.00;200.00]	152.00 [67.00;164.00] vs 194.0 [22.00;200.00]	No Data	.810
	**IL-6**	24.1[17.50;355.05] vs 35.00 [12.00;86.00]	25.00 [13.00;102.00] vs 35.00 [12.00;86.00]	No Data	.928
**Cluster #3**	**HBP**	110.50 [39.00;300.00] vs 104.5[31.00;223.25]	39.00 [33.00;91.00] vs 104.5[31.00;223.25]	35.00 [21.50;65.50] vs 104.5[31.00;223.25]	.127
	**SAA**	240.45[88.95;300.00] vs 280.0[123.40;300.00]	295.3[169.00;300.00] vs 280.0 [123.40;300.00]	300.0[33.50;300.00] vs 280.0 [123.40;300.00]	.375
	**CRP**	149.52 [49.81;200.00] vs 128.10 [45.40;200.00]	200.0[165.50;200.00] vs 128.1[45.40;200.00]	138.00 [52.00;169.50] vs 128.1[45.40;200.00]	.144
	**IL-6**	31.00 [12.00;62.75] vs 35.15 [16.00;52.73]	59.10 [53.50;194.50] vs 35.15 [16.00;52.73]	37.00 [16.00;145.50] vs 35.15 [16.00;52.73]	.856
**Cluster #4**	**HBP**	52.00 [12.00;113.00] vs 28.00 [16.00;90.00]	32.50 [17.25;104.00] vs 28.00 [16.00;90.00]	40.00 [13.00;87.50] vs 28.00 [16.00;90.00]	.988
	**SAA**	233.3[159.00;300.00] vs 246.5 [98.40;300.00]	253.40 [44.50;300.00] vs 246.5[98.40;300.00]	260.50 [58.25;300.00] vs 246.5 [98.40;300.00]	.991
	**CRP**	179.4 [112.00;200.00] vs 139.02 [93.18;200.00]	38.76 [13.50;130.75] vs 139.02 [93.18;200.00]	154.50 [28.00;200.00] vs 139.02 [93.18;200.00]	.303
	**IL-6**	47.50 [29.75;68.70] vs 61.50 [20.50;119.25]	27.30 [15.00;40.50] vs 61.50 [20.50;119.25]	50.50 [13.00;68.50] vs 61.50 [20.50;119.25]	.510
